# Cannabis use among Dutch patients with a primary brain tumor

**DOI:** 10.1093/nop/npaf009

**Published:** 2025-01-23

**Authors:** Vera Belgers, Niek A Rietveld, Philip C de Witt Hamer, Johanna M Niers

**Affiliations:** Department of Neurology, Amsterdam UMC location Vrije Universiteit Amsterdam, Amsterdam, The Netherlands; Cancer Center Amsterdam, Brain Tumor Center, Amsterdam, The Netherlands; Department of Neurosurgery, Amsterdam UMC location Vrije Universiteit Amsterdam, Amsterdam, The Netherlands; Department of Neurosurgery, Amsterdam UMC location Vrije Universiteit Amsterdam, Amsterdam, The Netherlands; Cancer Center Amsterdam, Brain Tumor Center, Amsterdam, The Netherlands; Department of Neurology, Amsterdam UMC location Vrije Universiteit Amsterdam, Amsterdam, The Netherlands; Cancer Center Amsterdam, Brain Tumor Center, Amsterdam, The Netherlands

**Keywords:** cannabidiol, glioma, marijuana, meningioma, Δ^9^-tetrahydrocannabinol

## Abstract

**Background:**

Cancer patients commonly use cannabis to improve symptoms or for presumed anticancer effects. However, the extent and type of cannabis use among Dutch primary brain tumor patients, along with their motivations, remains unclear. This study aims to determine the prevalence of prior or current cannabis use in patients with a primary brain tumor, the type of cannabis used, their motivation, how they perceived the effects of the cannabis, and what adverse effects they noticed.

**Methods:**

We conducted a cross-sectional survey of adult primary brain tumor patients who visited the neuro-oncology outpatient clinic at Amsterdam UMC between August and October 2023.

**Results:**

Of 100 responding patients, 51% had ever used cannabis and 14% currently used cannabis. Of the total group, 19% currently or previously used cannabis for tumor-related reasons, including symptom relief (*n* = 14; 74%) and presumed effect on the tumor (*n* = 8; 42%), indicating that some patients used it for multiple therapeutic purposes. Patients favored cannabidiol (CBD) over Δ^9^-tetrahydrocannabinol (THC). Self-reported sleep, anxiety, worrying, and depressive symptoms most frequently improved due to cannabis use, and the most common self-reported adverse events included drowsiness, dry mouth, and dizziness.

**Conclusions:**

A considerable amount of primary brain tumor patients use cannabis, often for symptom relief or presumed antitumor effects. The user rate underscores a demand for clinical research into the therapeutic efficacy of cannabis, particularly concerning its impact on symptoms like sleep disturbances, depressive symptoms, and anxiety/worrying, as well as its tumor-inhibiting capabilities.

**Key Points**

- Neuro-oncology patients use cannabis for symptom relief or antitumor effects.
- Cannabis improved self-reported sleep, anxiety, and depressive symptoms most often.

**Importance of the Study**

Patients with a primary brain tumor sometimes use (a form of) cannabis on their own initiative. In this study, we explore how often Dutch brain tumor patients use cannabis, in what forms and doses, and for what reasons. Understanding their motivations and experiences could help raise awareness among healthcare providers, foster open discussions, and better address patients’ needs.

Glioma patients frequently experience a wide array of symptoms, which are often not completely alleviated by current treatment of symptoms. This might lead them to explore alternative therapies, such as cannabis. Medical cannabis is cannabis with the purpose of relief of symptoms or inhibition of tumor growth, and it is popular among cancer patients.^1^ Similar to other cancer patients, a study in Florida showed that among 73 glioma patients, 32.8% turned to cannabis after diagnosis, and 70.8% of these patients currently still used cannabis.^2^ Main reasons for use included pain relief, nausea relief, appetite stimulation, relaxation, and to cope emotionally. However, the effects of cannabis on glioma-related symptoms remain insufficiently understood. Moreover, its antitumor effects have not been proven, specifically not in glioma patients.^3,4^

Cannabis consists of 2 main components: Δ^9^-tetrahydrocannabinol (THC) and cannabidiol (CBD), with distinct effects.^5^ THC can induce psychotropic effects typically associated with cannabis, such as distortions of vision, mental confusion, feeling dreamy, and difficulty in thinking. CBD shows potential to reduce seizure frequency in certain epilepsy syndromes and may have anxiolytic, sleep-enhancing, and analgesic properties.^5^

In the Netherlands, medical cannabis can be prescribed by healthcare providers and is distributed through certain licensed pharmacies. Moreover, cannabis for personal use is tolerated: THC can be obtained through dedicated cannabis shops, CBD is also available in drug stores.^6^ In the general Dutch population, 6% has used cannabis during the prior month.^7^ Due to the differences in cannabis legislation, cannabis use among glioma patients could be different from countries such as the USA.

Understanding the extent and the reason of cannabis use among Dutch primary brain tumor patients might raise awareness among healthcare providers and facilitate discussions about its efficacy and its safety. Moreover, understanding what type of cannabis patients use—ie, CBD- vs THC-dominant product—and how they perceive its effects might help in better understanding the needs and preferences of the brain tumor population and could guide future research endeavors that are most relevant to patients. Therefore, in this cross-sectional study, we explored the prevalence of prior or current cannabis use in patients with a primary brain tumor, the type of cannabis used, their motivation, how they perceived the effects of the cannabis, and what adverse effects they noticed.

## Methods

In this survey study, primary brain tumor patients physically attending the neuro-oncology outpatient clinic between August and October 2023 could be included. The medical ethics committee waived this study for the Medical Research Involving Human Subjects Act. All participants signed informed consent. Eligibility criteria included patients aged 18 years or older with either a radiological suspicion or a confirmed diagnosis of a primary brain tumor at any phase of the disease. Patients who were unable to read or understand Dutch were excluded. Those unable to complete the questionnaire for another reason, such as a significant language impairment or an inability to write or inability to visit the outpatient clinic, did not participate. Questionnaires could not be filled out by a proxy.

### Survey

We designed a custom cannabis use questionnaire to describe the demographics of cannabis users, time since diagnosis and questionnaire, their motivations for starting or stopping cannabis, the self-reported effects and adverse events, the predominant cannabis constituents THC or CBD, administration route, and dose (see Supplementary Material). All questions were multiple choice with the option to choose multiple answers, and a blank space for additional comments, see Supplementary Material for the questionnaire. Patients could report multiple reasons for cannabis use, and multiple administration routes. In selecting questions, we prioritized relevance and brevity, deriving insights from other cannabis-related surveys.^8–11^

### Descriptive Statistics

We performed all analyses in R v4.2.1. Descriptive statistics are displayed in mean and SD for normally distributed data as determined through Shapiro–Wilk tests. The result groups displayed in the table are mutually exclusive unless explicitly otherwise. Patients who reported using cannabis for presumed symptom relief or antitumor effects were categorized as tumor-related users, those using cannabis solely for recreational reasons as recreational users. “Never users” refers to people who had never used cannabis. “Current recreational users” (CRU) refers to patients using cannabis exclusively for recreational purposes at the time of assessment, while “prior recreational users” (PRU) had used cannabis for recreational purposes in the past but were no longer using it. “Current tumor-related users” (CTU) were individuals using cannabis at the time of the survey for tumor-related reasons, such as symptom relief or presumed antitumor effects. “Prior tumor-related users” (PTU) were those who had previously used cannabis for tumor-related reasons but had since stopped. The “total current users” included all current users, regardless of reason (CRU + CTU), and the “total tumor-related users” consisted of all patients who had ever used cannabis for tumor-related purposes (CTU + PTU).

## Results

The questionnaire was completed by 102 people. We excluded 2 people with a secondary brain tumor, resulting in a total of 100 patients, of whom 79% had a glioma diagnosis and 11% had a meningioma. Fifty-one (51%) patients had used cannabis during their lifetime, with 14% currently using cannabis. Of the complete group, 19% currently or previously used cannabis for tumor-related reasons, including symptom relief (*n* = 14; 74%) and presumed effect on the tumor (*n* = 8; 42%), indicating that some patients (3 patients; 16%) used it for multiple therapeutic purposes. Mean age for never-users was 54 years old (SD 14), for current recreational users 31 years old (SD 6.6), and for current tumor-related users 57 years old (SD 11). See Table 1 for all demographics.

**Table 1.** Demographics of Included Patients

		Recreational users (*N* = 32; 32%)		Tumor-related users (*N* = 19; 19%)			Total current and tumor-related users (*N* = 22; overlapping groups)	
	Never user (*N* = 49)	Current user (*N* = 3; CRU)	Prior user (*N* = 29; PRU)	Current user (*N* = 11; CTU)	Prior user (*N* = 8; PTU)	Total (*N* = 100)	Total current users (*N* = 14; 14%; CRU + CTU)	Total tumor-related users (*N* = 19; 19%; CTU + PTU)
**Age (years)**								
Mean (SD)	54 (± 14)	31 (± 6.6)	47 (± 13)	57 (± 11)	49 (± 19)	51 (± 14)	51 (± 15)	54 (± 15)
**Age groups**								
<40 years	8 (16%)	3 (100%)	10 (34%)	1 (9%)	3 (38%)	25 (25%)	4 (29%)	4 (21%)
40–60 years	24 (49%)	0 (0%)	14 (48%)	7 (64%)	3 (38%)	48 (48%)	7 (50%)	10 (53%)
>60 years	17 (35%)	0 (0%)	5 (17%)	3 (27%)	2 (25%)	27 (27%)	3 (21%)	5 (26%)
**Sex**								
Female	23 (47%)	2 (67%)	9 (31%)	7 (64%)	5 (62%)	46 (46%)	9 (64%)	12 (63%)
Male	26 (53%)	1 (33%)	20 (69%)	4 (36%)	3 (38%)	54 (54%)	5 (36%)	7 (37%)
**Tumor type**								
Glioma, grade 1	2 (4%)	0 (0%)	3 (10%)	0 (0%)	1 (12%)	6 (6%)	0 (0%)	1 (5 %)
Glioma, grade 2	13 (27%)	2 (67%)	8 (28%)	5 (45%)	0 (0%)	28 (28%)	7 (50%)	5 (26%)
Glioma, grade 3	10 (20%)	0 (0%)	8 (28%)	1 (9%)	2 (25%)	21 (21%)	1 (7%)	3 (16%)
Glioma, grade 4	11 (22%)	0 (0%)	6 (21%)	3 (27%)	4 (50%)	24 (24%)	3 (21%)	7 (37%)
Meningioma	8 (16%)	0 (0%)	2 (7%)	1 (9%)	0 (0%)	11 (11%)	1 (7%)	1 (5%)
Other	4 (8%)	1 (33%)	2 (7%)	0 (0%)	1 (12%)	8 (8%)	1 (7%)	1 (5%)
Suspect for brain tumor	1 (2%)	0 (0%)	0 (0%)	1 (9%)	0 (0%)	2 (2%)	1 (7%)	1 (5%)
**Time since diagnosis at time of questionnaire**								
<1 year	7 (14%)	1 (33%)	5 (17%)	5 (45%)	1 (12%)	19 (19%)	6 (43%)	6 (32%)
1–3 year	14 (29%)	1 (33%)	5 (17%)	1 (9%)	0 (0%)	21 (21%)	2 (14%)	1 (5%)
3–5 year	7 (14%)	1 (33%)	7 (24%)	1 (9%)	2 (25%)	18 (18%)	2 (14%)	3 (16%)
>5 year	19 (39%)	0 (0%)	12 (41%)	4 (36%)	5 (62%)	40 (40%)	4 (29%)	9 (47%)
Missing	2 (4.1%)	0 (0%)	0 (0%)	0 (0%)	0 (0%)	2 (2%)	0 (0%)	0 (0%)
**Current treatment**								
None	40 (82%)	2 (67%)	18 (62%)	8 (73%)	5 (62%)	73 (73%)	10 (71%)	13 (68%)
Surgery soon	0 (0%)	0 (0%)	2 (7%)	0 (0%)	0 (0%)	2 (2%)	0 (0%)	0 (0%)
Chemotherapy	4 (8%)	1 (33%)	5 (17%)	2 (18%)	1 (12%)	13 (13%)	3 (21%)	3 (16%)
Radiation	3 (6%)	0 (0%)	0 (0%)	0 (0%)	0 (0%)	3 (3%)	0 (0%)	0 (0%)
Chemoradiation	1 (2%)	0 (0%)	2 (7%)	1 (9%)	2 (25%)	6 (6%)	1 (7%)	3 (16%)
Other	0 (0%)	0 (0%)	1 (3%)	0 (0%)	0 (0%)	1 (1%)	0 (0%)	0 (0%)
Missing	1 (2%)	0 (0%)	1 (3%)	0 (0%)	0 (0%)	2 (2%)	0 (0%)	0 (0%)

CRU, current recreational user; CTU, current tumor-related user; PRU, prior recreational user; PTU, prior tumor-related user.

Table 2 displays details on cannabis use in prior and current users. Of the current cannabis users, most patients (*n* = 9; 64%) used it daily, 57% (*n* = 8) used it for symptom relief and 43% (*n* = 6) used it for presumed effects on their tumor. Tumor-related prior or current users mostly used oral forms of cannabis (*n* = 15; 79%). Of patients using cannabis for tumor-related reasons, 21% (*n* = 4) were unaware of the components of their cannabis. Patients (had) used doses ranging from 12 to 320 mg CBD and between 60 mg THC and 0.5–5 joints per day, whilst 42% (*n* = 8) of current users did not know the dose they were using (data not shown; results based on a combination of questions 11 and 13 regarding cannabis components and dose).

**Table 2.** Details on Cannabis Use in Current or Prior Cannabis Users

	Recreational users (*N* = 32; 63%)		Tumor-related users (*N* = 19; 37%)			Total current and tumor-related users (*N* = 22; overlapping groups)	
	Current user (*N* = 3; CRU)	Prior user (*N* = 29; PRU)	Current user (*N* = 11; CTU)	Prior user (*N* = 8; PTU)	Total (*N* = 51)	Total current users (*N* = 14; 27%; CRU + CTU)	Total tumor-related users (*N* = 19; 37%; CTU + PTU)
**First time using cannabis**							
Before diagnosis	3 (100%)	29 (100%)	6 (55%)	6 (75%)	44 (86%)	9 (64%)	12 (63%)
After diagnosis	0 (0%)	0 (0%)	5 (45%)	2 (25%)	7 (14%)	5 (36%)	7 (37%)
**Frequency of current cannabis use**							
Daily	1 (33%)		8 (73%)		9 (18%)	9 (64%)	8 (42%)
Multiple times per week	0 (0%)		1 (9%)		1 (2%)	1 (7%)	1 (5%)
Once per week	1 (33%)		2 (18%)		3 (6%)	3 (21%)	2 (11%)
Once per month	1 (33%)		0 (0%)		1 (2%)	1 (7%)	0 (0%)
NA	0 (0%)		0 (0%)		37 (73%)	0 (0%)	8 (42%)
**Method of cannabis use**							
Capsules or tablets	0 (0%)	0 (0%)	2 (18%)	0 (0%)	2 (4%)	2 (14%)	2 (11%)
Drops	0 (0%)	0 (0%)	6 (55%)	7 (88%)	13 (25%)	6 (43%)	13 (68%)
Drops and smoking	0 (0%)	1 (3%)	0 (0%)	0 (0%)	1 (2%)	0 (0%)	0 (0%)
Smoking or other	3 (100%)	28 (97%)	3 (27%)	1 (12%)	35 (69%)	6 (43%)	4 (21%)
**Cannabis components**							
Only or mainly THC	1 (33%)	10 (34%)	3 (27%)	1 (12%)	15 (29%)	4 (29%)	4 (21%)
Equal CBD/THC	0 (0%)	0 (0%)	1 (9%)	0 (0%)	1 (2%)	1 (7%)	1 (5%)
Only or mainly CBD	0 (0%)	0 (0%)	5 (45%)	5 (62%)	10 (20%)	5 (36%)	10 (53%)
Don’t know	1 (33%)	19 (66%)	2 (18%)	2 (25%)	24 (47%)	3 (21%)	4 (21%)
Missing	1 (33%)	0 (0%)	0 (0%)	0 (0%)	1 (2.0%)	1 (7%)	0 (0%)
**Reasons for cannabis use**							
Effects on tumor growth	0 (0%)	0 (0%)	3 (27%)	2 (25%)	5 (10%)	3 (21%)	5 (26%)
Effects on tumor and symptom relief	0 (0%)	0 (0%)	3 (27%)	0 (0%)	3 (6%)	3 (21%)	3 (16%)
Recreational	3 (100%)	29 (100%)	0 (0%)	0 (0%)	32 (63%)	3 (21%)	0 (0%)
Recreational and symptom relief	0 (0%)	0 (0%)	1 (9%)	0 (0%)	1 (2%)	1 (7%)	1 (5%)
Symptom relief	0 (0%)	0 (0%)	4 (36%)	6 (75%)	10 (20%)	4 (29%)	10 (53%)
**Reasons for stopping**							
Concerns about adverse events		5 (17%)		0 (0%)	5 (10%)		0 (0%)
Costs		0 (0%)		1 (12%)	1 (2%)		1 (5%)
No effects		3 (10%)		3 (38%)	6 (12%)		3 (16%)
Missing/other/NA		21 (72%)		4 (50%)	39 (77%)		15 (79%)

CRU, current recreational user; CTU, current tumor-related user; NA, not applicable; PRU, prior recreational user; PTU, prior tumor-related user.

Among prior users (*n* = 37), 25 people (68%) did not select one of the provided multiple choice options for stopping cannabis. Four individuals (11%) did not provide any reason, while 18 (49%) stated a form of “no longer interested,” one person stopped due to their job, another due to their diagnosis, and one reported they “simply stopped” (data not shown).

**Figure 1.**
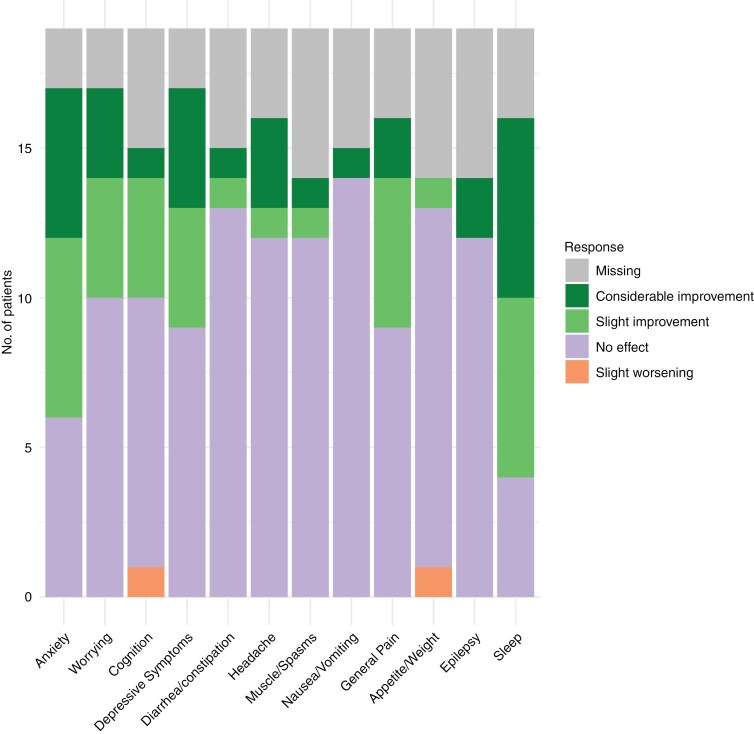
Self-reported effects of cannabis in prior and current tumor-related cannabis users.

**Figure 2.**
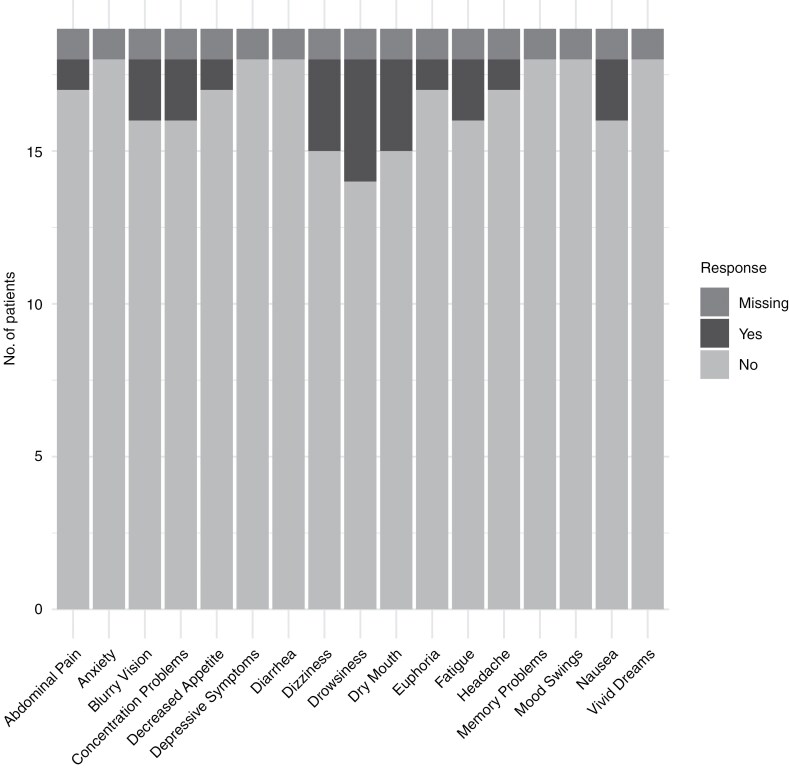
Self-reported adverse events of cannabis in prior and current tumor-related cannabis users.

For tumor-related users, the symptoms that were reported as most frequently improved through cannabis were sleep, anxiety, worrying, and depressive symptoms, see Figure 1. Only 2 symptoms (cognition and appetite) worsened a little as reported by one person, no people reported a substantial worsening of symptoms. Some patients did not respond to certain symptom-related questions, with nonresponses ranging from to individuals for the anxiety, worrying, and depressive symptoms items to 5 people for the items on muscle/spasms, appetite/weight, and epilepsy. The most frequently reported adverse events were drowsiness, dry mouth, and dizziness, see Figure 2. One person did not respond to the adverse event question.

## Discussion

In this study of Dutch primary brain tumor patients, a little over half of patients reported past or current cannabis use, with 14% actively using it. Of all brain tumor patients currently using cannabis, 57% used it for symptom relief and 43% used it for presumed effects on their tumor.

The percentage of current cannabis users in our cohort was comparable to that of Dutch cancer patients undergoing systemic anticancer treatment,^12^ but higher than in the general population. In 2021, an estimated 1.3% of European adults aged 25–64 used cannabis daily, and in the Netherlands, 6.4% of adults used it in the last month.^7^ Cannabis use in the Netherlands seems less prevalent than in the USA, where 14% of adults aged 26 or older used cannabis in the past month.^13^ Similar to these differences in the general population, the percentage of current cannabis users was lower in our cohort than among 73 glioma patients in Florida, where 22% currently used cannabis.^2^ Although cannabis use among Dutch patients seems less prevalent than in the USA, cannabis users still comprise a significant portion of patients.

In a comparable German study of 102 patients with an intracranial glioma, 14% used alternative therapies, although it is unclear if this included cannabis or other substances.^14^ Another survey among 277 French glioma patients found that 9% used medication without a prescription, and 22% used a food supplement.^15^ The authors did not specify the nature of these substances, but cannabis could potentially fall under either category. To our knowledge, no specific data exist on the use general complementary therapies among Dutch neuro-oncology patients.

Interestingly, a significant percentage of patients, including tumor-related users, first used cannabis before their tumor diagnosis. This could be attributed to the more relaxed regulations in the Netherlands and the high rate of lifetime cannabis experimentation, which stands at 28.8% in the general population.^6^ It is plausible that patients who had previously tried cannabis were more open to using it again following their brain tumor diagnosis.

Of all brain tumor patients currently using cannabis, most people use it for symptom relief. The most commonly reported improvements were in sleep, anxiety, worrying, and depressive symptoms. Patients reported cannabis use to be beneficial with one patient reporting only a little worsening of cognition and appetite, whereas most other symptoms either improved a little or improved a lot. Of note, some answers were missing. Possibly, patients did not experience a specific symptom such as epilepsy or cognitive problems prior to starting cannabis so therefore did not know cannabis’ effect on it, did not remember the effect if they were prior users, or had a lack of time as they completed the questionnaire in the waiting room. Nevertheless, our findings align with the findings in the Florida glioma cohort, where patients also reported an improved ability to cope emotionally, to relax, and to sleep. However, that study only reported 7 symptoms and they did not report if any symptoms worsened.^2^

Our findings on perceived improvements in sleep corroborate a recent meta-analysis in patients with sleep disturbances, which found modest evidence for small benefits of cannabis on sleep quality and sleep disturbance among chronic pain patients.^16^ However, they did not differentiate between different cannabis components.

Additionally, patients in our cohort frequently reported improvements in mental health symptoms, ie, anxiety, worrying, and depressive symptoms. This corroborates a 2019 meta-analysis among patients with various diseases^17^: THC did not affect depressive symptoms but improved anxiety, though the evidence was graded low-quality and the effects were small. Conversely, another meta-analysis shows that acute administration of THC induced both anxiety and depression in healthy volunteers.^18^ CBD, however, shows less conflicting results and might be promising in the management of anxiety based on animal research and some small studies in healthy volunteers and patients with an anxiety disorder.^19^

A significant part of current cannabis users also reported using it for presumed effects on their tumor. This is more than in the Florida glioma cohort, where only 3 out of 73 patients independently reported this reason,^2^ but aligns with a Dutch study involving 153 patients with extra-cranial cancer, where 46% used cannabis for presumed antitumor effects.^12^

While some preclinical studies have suggested potential benefits on cell growth, apoptosis, and angiogenesis, clinical evidence remains insufficient with just one placebo-controlled clinical trial.^20,21^ In the randomized, double-blind, and placebo-controlled part of this phase 1b study involving 21 patients with a recurrent glioblastoma, a CBD:THC spray alongside Temozolomide resulted in a more favorable survival.^21^ However, the small sample size, favorable demographics, 2 early deaths in the placebo group, and unavailable molecular information complicate the interpretation of the results.^4,21^

In our cohort, few adverse events were reported, with the most commonly reported being drowsiness, dry mouth, and dizziness. Given the observational design of our study, the self-reported adverse events could possibly be attributed to other medications or treatments. Moreover, 21% of patients were undergoing chemotherapy at the time of questionnaire completion, and 7% were receiving chemoradiation, which may have also contributed to these reported adverse events. However, these findings are consistent with known adverse events of medical cannabis, which are common and include somnolence, dizziness, dry mouth, and nausea.^22^ The occurrence of adverse events might depend on various aspects such as the cannabis components and dosage of cannabis used. The few self-reported adverse events in our study could be due to the preferred use of CBD among participants. CBD has a more favorable adverse event profile than THC with few serious adverse events, and is generally well-tolerated.^23^

We found that a considerable proportion of patients were unaware of the constituents of their cannabis. Of the patients using cannabis for tumor-related effects who did know the constituents, the majority favored CBD-dominant products. Doses of cannabis varied greatly among those who knew the dose of their cannabis product. These findings underscore the need for more comprehensive information regarding the distinct effects of both CBD and THC and of different doses.^5^

### Clinical Implications

We show that a considerable amount of patients with a primary brain tumor use cannabis or have used cannabis. Patients were generally very positive about the effects. However, the cross-sectional nature of this study withholds us from making any firm conclusions about the effects of cannabis on various symptoms, with potential recall bias, patients’ expectations, placebo effects, concurrent other therapies, and an unknown baseline level of symptoms possibly affecting perceptions of its efficacy. In the Netherlands, healthcare providers can prescribe cannabis. Nevertheless, there is no convincing evidence for the efficacy on either symptom relief or tumor inhibition. As with every therapy, we would advise against its prescription until high-quality evidence is available. The significant percentage of brain tumor patients using cannabis and the reported beneficial effects reflect a clear need for more reliable evidence regarding the effect of cannabis on symptom relief, specifically anxiety/worrying, sleep and depressive symptoms, and tumor growth. Fortunately, recent initiatives have begun to address this gap.^4,24–27^

Given the prevalence rate of cannabis use among patients, it may be advisable for healthcare providers to actively inquire about cannabis use and consider screening for potential side effects, such as elevated liver enzymes, particularly in patients using CBD, as was the case for the majority of current users. Additionally, if cannabis use is reported, it would be beneficial to discuss the current, albeit limited, evidence and further explore the patient’s needs and motivations.

### Strengths and Limitations

To our knowledge, this is the first Dutch study assessing cannabis use in the brain tumor population. However, the sample size withheld us from characterizing typical cannabis users. Additionally, we did not report responder rate. The significant percentage of patients who never used cannabis makes a possible selection bias more unlikely, especially since it was our impression that all patients who had the time were willing to participate. Nonetheless, we cannot fully exclude the possibility of responder bias. Also, the survey was self-reported, and we did not verify details such as cannabis components, dosages, or current treatment. Information on disease phase was also lacking and can only be partly inferred from current treatment data. Moreover, detailed information on type of chemotherapy and other possibly relevant information such as other medication use such as antiseizure medication, was not collected. Furthermore, recall bias might play a role specifically in prior tumor-related cannabis users.

## Conclusion

A considerable proportion of Dutch people with a primary brain tumor use cannabis, with the most frequently reported reasons being symptom relief and presumed beneficial effects on tumor growth. The user rate reflects the patients’ needs for randomized controlled trials into the effects of cannabis, mainly on sleep, mental health, and tumor effects.

## Supplementary material

Supplementary material is available online at *Neuro-Oncology Practice* (https://academic.oup.com/nop/).

npaf009_suppl_Supplementary_Material

## Data Availability

Data of this study is not publicly available due to privacy regulations.

## References

[1] Osaghae I , Chido-Amajuoyi OG, Talluri R, Shete S. Prevalence, reasons for use, perceived benefits, and awareness of health risks of cannabis use among cancer survivors – implications for policy and interventions [published online ahead of print December 29, 2023]. J Cancer Surviv. doi: https://doi.org/10.1007/s11764-023-01526-7. PMC11817783 38158514

[2] Reblin M , Sahebjam S, Peeri NC, et al Medical cannabis use in glioma patients treated at a comprehensive cancer center in Florida. J Palliat Med. 2019;22(10):1202–1207. 31081711 10.1089/jpm.2018.0528 PMC7364313

[3] National Academies of Sciences, Engineering, and Medicine; Health and Medicine Division; Board on Population Health and Public Health Practice; Committee on the Health Effects of Marijuana: An Evidence Review and Research Agenda. Therapeutic Effects of Cannabis and Cannabinoids. The Health Effects of Cannabis and Cannabinoids: The Current State of Evidence and Recommendations for Research. Washington (DC): National Academies Press (US); 2017:85–140. 28182367

[4] Doherty GJ , de Paula BHR. Cannabinoids in glioblastoma multiforme—hype or hope? Br J Cancer. 2021;124(8):1341–1343. 33623077 10.1038/s41416-021-01265-5 PMC8039024

[5] Stella N. THC and CBD: similarities and differences between siblings. Neuron. 2023;111(3):302–327. 36638804 10.1016/j.neuron.2022.12.022 PMC9898277

[6] Nationale Drug Monitor. Wetgeving, beleid en preventie: 2.1.4 medicinale cannabis. trimbos-instituut, Utrecht & WODC, Den Haag. 2024 Available at https://www.nationaledrugmonitor.nl/wetgeving-en-beleid-medicinale-cannabis/. Accessed January 17, 2024.

[7] European Monitoring Centre for Drugs and Drug Addiction. European drug report 2023: trends and developments. 2023 Available at https://www.euda.europa.eu/publications/european-drug-report/2023_en. Accessed January 17, 2024.

[8] Lintzeris N , Mills L, Abelev SV, et al Medical cannabis use in Australia: consumer experiences from the online cannabis as medicine survey 2020 (CAMS-20). Harm Reduct J. 2022;19(1):1–10. 35907959 10.1186/s12954-022-00666-w PMC9338505

[9] Lapham GT , Matson TE, Bobb JF, et al Prevalence of cannabis use disorder and reasons for use among adults in a us state where recreational cannabis use is legal. JAMA Netw Open. 2023;6(8):e2328934. 37642968 10.1001/jamanetworkopen.2023.28934 PMC10466162

[10] Nielsen SW , Ruhlmann CH, Eckhoff L, et al Cannabis use among Danish patients with cancer: a cross-sectional survey of sociodemographic traits, quality of life, and patient experiences. Support Care Cancer. 2022;30(2):1181–1190. 34453567 10.1007/s00520-021-06515-z

[11] Pergam SA , Woodfield MC, Lee CM, et al Cannabis use among patients at a comprehensive cancer center in a state with legalized medicinal and recreational use. Cancer. 2017;123(22):4488–4497. 28944449 10.1002/cncr.30879 PMC5698756

[12] Oelen Y , Revenberg S, de Vos-Geelen J, et al Cannabinoid consumption among cancer patients receiving systemic anti-cancer treatment in the Netherlands. J Cancer Res Clin Oncol. 2023;149(5):1863–1872. 35779108 10.1007/s00432-022-04085-z PMC10097765

[13] Substance Abuse and Mental Health Services Administration. Key substance use and mental health indicators in the united states: results from the 2022 National survey on drug use and health (HHS Publication No. PEP23-07-01-006, NSDUH Series H-58). 2023 Available at https://www.samhsa.gov/data/sites/default/files/reports/rpt42731/2022-nsduh-nnr.pdf. Accessed January 17, 2024.

[14] Ottenhausen M , Renovanz M, Bartz I, et al Use of complementary therapies and supportive measures of patients with intracranial gliomas—a prospective evaluation in an outpatient clinic. J Neurooncol. 2024;168(3):507–513. 38709354 10.1007/s11060-024-04696-1 PMC11186898

[15] Le Rhun E , Devos P, Bourg V, et al Complementary and alternative medicine use in glioma patients in France. J Neurooncol. 2019;145(3):487–499. 31637628 10.1007/s11060-019-03315-8

[16] AminiLari M , Wang L, Neumark S, et al Medical cannabis and cannabinoids for impaired sleep: a systematic review and meta-analysis of randomized clinical trials. Sleep. 2022;45(2):zsab234. 34546363 10.1093/sleep/zsab234

[17] Black N , Stockings E, Campbell G, et al Cannabinoids for the treatment of mental disorders and symptoms of mental disorders: a systematic review and meta-analysis. Lancet Psychiatry. 2019;6(12):995–1010. 31672337 10.1016/S2215-0366(19)30401-8 PMC6949116

[18] Hindley G , Beck K, Borgan F, et al Psychiatric symptoms caused by cannabis constituents: a systematic review and meta-analysis. Lancet Psychiatry. 2020;7(4):344–353. 32197092 10.1016/S2215-0366(20)30074-2 PMC7738353

[19] García-Gutiérrez MS , Navarrete F, Gasparyan A, et al A potential new alternative for the treatment of anxiety, depression, and psychotic disorders. Biomolecules. 2020;10(11):1575. 33228239 10.3390/biom10111575 PMC7699613

[20] Rodriguez-Almaraz JE , Butowski N. Therapeutic and supportive effects of cannabinoids in patients with brain tumors (CBD oil and cannabis). Curr Treat Options Oncol. 2023;24(1):30–44. 36633803 10.1007/s11864-022-01047-y PMC9867687

[21] Twelves C , Sabel M, Checketts D, et al; GWCA1208 study group. A phase 1b randomised, placebo-controlled trial of nabiximols cannabinoid oromucosal spray with temozolomide in patients with recurrent glioblastoma. Br J Cancer. 2021;124(8):1379–1387. 33623076 10.1038/s41416-021-01259-3 PMC8039032

[22] Pratt M , Stevens A, Thuku M, et al Benefits and harms of medical cannabis: a scoping review of systematic reviews. Syst Rev. 2019;8(1):1–35. 31823819 10.1186/s13643-019-1243-x PMC6905063

[23] Chesney E , Oliver D, Green A, et al Adverse effects of cannabidiol: a systematic review and meta-analysis of randomized clinical trials. Neuropsychopharmacology. 2020;45(11):1799–1806. 32268347 10.1038/s41386-020-0667-2 PMC7608221

[24] Gruber S. A clinical trial of a hemp-derived, high cannabidiol product for anxiety in glioblastoma patients. Identifier: NCT05753007. 2023 Available at https://classic.clinicaltrials.gov/ct2/show/NCT05753007. Accessed January 17, 2024.

[25] Belgers V. Glioma: reducing anxiety by conSuming cannabinoidS – GRASS study. Identifier NL9623. 2024 Available at https://onderzoekmetmensen.nl/en/trial/20540. Accessed January 17, 2024.

[26] Leaf Vertical Inc. A study of the efficacy of cannabidiol in patients with multiple myeloma, glioblastoma multiforme, and GI malignancies. Identifier: NCT03607643. 2018 Available at https://clinicaltrials.gov/study/NCT03607643. Accessed January 17, 2024.

[27] Bhaskaran D , Savage J, Patel A, et al A randomised phase II trial of temozolomide with or without cannabinoids in patients with recurrent glioblastoma (ARISTOCRAT): protocol for a multi-centre, double-blind, placebo-controlled trial. BMC Cancer. 2024;24(1):83. 38225549 10.1186/s12885-023-11792-4 PMC10790538

