# Supplementary material for: Cannabis use among Dutch patients with a primary brain tumor
Source: Neurooncol Pract. 2025 Jan 23;12(4):714–22. doi: 10.1093/nop/npaf009 (PMC12349755; doi:10.1093/nop/npaf009)
Supplement: npaf009_suppl_Supplementary_Material [file npaf009_suppl_supplementary_material.docx]

**Supplementary material: Cannabis Use Questionnaire for Patients with a Brain Tumor (translated from Dutch)**

**Cannabis Use Questionnaire for Patients with a Brain Tumor (translated from Dutch)**

Study Number (by researcher):______

*1. How old are you?* _________________ years

*2. What is your gender?*

 Male  Female

 Prefer not to answer  Other, please specify:___________________

*3. Do you have or have you had a brain tumor?*

 Yes  No; end of questionnaire

*4. What kind of brain tumor was/is this?*

 Meningioma  Glioma grade 1

 Glioma grade 2  Glioma grade 3

 Glioma grade 4  Other; please specify:________________________

*5. How long ago was your brain tumor first diagnosed?*

 0 – 6 months  7 months – 1 year

 1 – 2 years  2 – 3 years

 3 – 4 years  4 – 5 years

 More than 5 years

*6. Are you currently receiving treatment for your brain tumor?*

 No

 No, but I will have surgery soon

 Yes; I am currently receiving radiation combined with chemotherapy

 Yes; I am currently receiving only radiation

 Yes; I am currently receiving only chemotherapy

 Other, please specify:________________________________________________

**CANNABIS USE**

The following section is about marijuana or cannabis use. By 'cannabis', we mean all products derived from the cannabis plant, including hash, THC products, and cannabidiol (CBD) products.

*7. Have you ever used cannabis?*

 Yes

 No; end of questionnaire

*8. When did you first use cannabis?*

 Before the brain tumor diagnosis

 After the brain tumor diagnosis

Optional explanation:____________________________________________________

*9. Are you currently using cannabis?*

 Yes  No; proceed to question 11

*10. How often do you currently use cannabis?*

 Daily  Several times a week

 Once a week  Once a month

 Less than once a month  Other, please specify:___________________

*11. What is the composition of the cannabis you use(d)?*

 THC only  Mainly THC, a little CBD

 CBD only  Mainly CBD, a little THC

 Equal CBD/THC  Don’t know

 Other, please specify:________________________________________________

*12. In what form do or did you take cannabis?*

 Drops/oil  Capsules or tablets

 Smoking via joints  Vaping via a vaporizer

 Smoking via waterpipe or bong  Spray

 Baked into food like cake  Tea

 Other, please specify:________________________________________________

*13. What is the dosage per day? ____________* tablets/joints/drops/other (circle) of *____________* mg/percent/other (circle)

*14. What is the main reason you use(d) cannabis?*

 Recreational (for fun)  For the effects on the tumor

 To reduce symptoms, specifically:______________________________________

*15.* *What effect did or does cannabis have on the following symptoms? Check the box:*

|  | Considerable improvement | Slight improvement | No effect | Slight worsening | Considerable worsening |
| --- | --- | --- | --- | --- | --- |
| Anxiety |  |  |  |  |  |
| Worrying |  |  |  |  |  |
| Cognition (such as memory, concentration) |  |  |  |  |  |
| Depressive symptoms |  |  |  |  |  |
| Diarrhea/ constipation |  |  |  |  |  |
| Headache |  |  |  |  |  |
| Muscle spasms |  |  |  |  |  |
| Nausea/ vomiting |  |  |  |  |  |
| Pain (other than headache) |  |  |  |  |  |
| Poor appetite/ weight loss |  |  |  |  |  |
| Epileptic seizures |  |  |  |  |  |
| Sleep |  |  |  |  |  |
| Other, please specify: |  |  |  |  |  |

*16. If you stopped using cannabis: why did you stop?*

 Too expensive  Too difficult to obtain

 It did not improve my symptoms

 I was worried about side effects

 Other, please specify:________________________________________________

*17. If you have or had side effects from cannabis, what are or were they? (check all that apply)*

 Diarrhea  Stomach or abdominal pain

 Dizziness  Blurry vision

 Memory issues  Decreased concentration

 Mood swings  Anxiety

 Depressive symptoms  Euphoria

 Dry mouth  Fatigue

 Vivid dreams  Headache

 Nausea/ vomiting  Drowsiness/ sedation

 Decreased appetite

 Other, please specify:________________________________________________
